# *Ginkgolic Acid* as a carbapenem synergist against KPC-2 positive *Klebsiella pneumoniae*

**DOI:** 10.3389/fmicb.2024.1426603

**Published:** 2024-08-21

**Authors:** Yuping Song, Yinuo Zou, Lei Xu, Jianfeng Wang, Xuming Deng, Yonglin Zhou, Dan Li

**Affiliations:** ^1^Department of Respiratory Medicine, Center for Pathogen Biology and Infectious Diseases, Key Laboratory of Organ Regeneration and Transplantation of the Ministry of Education, The First Hospital of Jilin University, Changchun, Jilin, China; ^2^State Key Laboratory for Diagnosis and Treatment of Severe Zoonotic Infectious Diseases, Key Laboratory for Zoonosis Research of the Ministry of Education, Institute of Zoonosis, and College of Veterinary Medicine, Jilin University, Changchun, China; ^3^Key Laboratory of Ministry of Education for Conservation and Utilization of Special Biological Resources in the Western China, School of Life Sciences, Ningxia University, Yinchuan, China

**Keywords:** KPC-2, *Ginkgolic Acid* (C13:0), carbapenems, *Klebsiella pneumoniae*, resistance

## Abstract

The successful evolution of KPC-2 in bacteria has limited the clinical practice of carbapenems. This dilemma deteriorated the prognosis of associated infections and hence attracted increasing attention from researchers to explore alternative therapeutic options. Here, the enzyme inhibition assay was first performed to screen for a potent KPC-2 inhibitor. The synergistic effect of the candidate with carbapenems was further confirmed by checkboard minimum inhibitory concentration (MIC) assay, time-killing assay, disk diffusion method, and live/dead bacteria staining analysis. The mechanisms by which the candidate acts were subsequently explored through molecular dynamics (MD) simulations, etc. Our study found that *Ginkgolic Acid* (C13:0) (GA) exhibited effective KPC-2 inhibitory activity in both laboratory strain and clinical strain containing KPC-2. It could potentiate the killing effect of carbapenems on KPC-2-positive *Klebsiella pnenmoniae(K. pnenmoniae)*. Further explorations revealed that GA could competitively bind to the active pocket of KPC-2 with meropenem (MEM) via residues Trp_104,_ Gly_235,_ and Leu_166_. The secondary structure and functional groups of KPC-2 were subsequently altered, which may be the main mechanism by which GA exerted its KPC-2 inhibitory effect. In addition, GA was also found to synergize with MEM to disrupt membrane integrity and increase membrane permeability, which may be another mechanism by which GA reinforced the bactericidal ability of carbapenems. Our study indicated that GA was a significant KPC-2 inhibitor that could prolong the lifespan of carbapenems and improve the prognosis of patients.

## 1 Introduction

Bacterial resistance, a phenomenon mainly attributed to the misuse and abuse of antibiotics, has affected approximately 22 countries around the world, triggering a widespread antibiotic resistance crisis (Cosgrove, 2006; Maragakis et al., 2008; de Kraker et al., 2011; Dos Santos et al., 2021). As the last resort against multidrug-resistant bacterial infections, the clinical efficacy of carbapenems has been greatly reduced with the emergence of carbapenem-resistant strains. Among these, carbapenem-resistant *Klebsiella pneumoniae* (CRKP) has attracted extreme attention by virtue of its high mortality rate and is already classified as a member of “ESKAPE” pathogen group (*Enterococcus faecium, Staphylococcus aureus, Klebsiella pneumoniae* (*K. pneumoniae*)*, Acinetobacter baumannii, Pseudomonas aeruginosa, and Enterobacter* spp; Xu et al., 2017). A continuous increase in the resistance rate of *K. pneumoniae* to carbapenems has been reported (Wu et al., 2021), posing a serious threat to human life and property. To address this dilemma, the research of new antibiotics or the seek for antibiotic synergists is imperative.

Carbapenem resistance can be caused by a variety of mechanisms, of which carbapenemase production is the most common in CRKP. *Klebsiella pneumoniae* carbapenemase-2 (KPC-2), a class A serine β-lactamase, can hydrolyze all FDA-approved β-lactam antibiotics and β-lactamase inhibitors (Munoz-Price et al., 2013; Pemberton et al., 2017), and is widely disseminated at an alarming rate in China (Zhang et al., 2017). In this critical situation, the pace of new antibiotic development lags far behind the emergence of antibiotic-resistant strains, forced by the enormous costs in time and money. However, restoring the susceptibility of KPC-2-positive *K. pneumoniae* to carbapenems appears to be a less costly and feasible strategy.

Accumulating evidence has demonstrated that enzyme inhibitors exhibited excellent efficacy on antibiotic sensitization without significant adverse effects (Xu et al., 2022; Zhou et al., 2022). Given the above theoretical basis, we performed a KPC-2-targeted biochemical screen to reverse antibiotic resistance. Herein, we discovered that *Ginkgolic Acid* (C13:0) (GA), a natural compound derived from *Ginkgo biloba*, synergized with carbapenems against KPC-2 positive bacteria as a KPC-2 inhibitor. Inspiringly, GA could simultaneously potentiate the interference of carbapenems on bacterial membranes thereby further reinforcing the bactericidal efficacy. Our findings provided a feasible and promising therapeutic strategy to combat intractable carbapenem-resistant *K. pneumoniae* infection.

## 2 Materials and methods

### 2.1 Bacterial strains and reagents

*E. coli* BL21(DE3) (pET28a-KPC-2) and *E. coli* BL21(DE3)-pET28a were constructed by our laboratory*. K. pneumoniae* ST-C1 and *K. pneumoniae* 43816 is a KPC-2 negative standard strain purchased from the American Type Culture Collection (ATCC). *K. pneumoniae* ST-C1 is a KPC-2-containing clinical strain. All the strains of *K. pneumoniae* were grown in LB broth (BIOFOUNT). The GA (dissolved in dimethyl sulfoxide) was bought from Chengdu Deruike Biotechnology Co., Ltd. Meropenem (MEM), imipenem, and kanamycin used in this study were purchased from the China Institute of Veterinary Drug Control. Nitrocefin (CAS: 41906-86-9) was obtained from TOKU-E Company, Bellingham, WA, United States.

### 2.2 Expression and purification of KPC-2 and its mutants

The expression vectors for the KPC-2 mutants were generated using a QuikChange site-directed mutagenesis kit (Stratagene, La Jolla, CA, United States) based on *E. coli* BL21(DE3) (pET28a) (KPC-2) constructed in our previous study (Zhou et al., 2020b), during which the gene-specific primers detailed in Supplementary Table 1 played an integral role. The purification of KPC-2 and its mutants was conducted successively after verifying the sequences of mutant strains by nucleotide sequencing.

### 2.3 Enzyme inhibition assay

The inhibitory effect of compounds on KPC-2 activity was determined by detecting the hydrolysis ability of β-lactamase against nitrocefin substrate as described previously (Zhou et al., 2020a). Filtered phosphate-buffered saline (PBS) was mixed with purified KPC-2 protein or bacterial supernatants containing KPC-2, and meanwhile different concentrations of *compounds* were added at a final concentration of 0–64 μg/mL. After incubation at 37°C for 15 min, diluted nitrocefin was added for further incubation. 25 min later, the change of solution in color and absorbance at 492 nm was observed at room temperature. The IC_50_ (half maximal inhibitory concentration) of the compound was calculated using GraphPad prism software.

### 2.4 Bacterial growth curve analysis

The growth curve analysis was performed to clarify the effect of GA on the proliferation of *K. pneumoniae* (Zhou et al., 2018). Briefly, bacteria cultured overnight were diluted into fresh LB medium at a ratio of 1:100. Then, a total of 150 mL of *K. pneumoniae* (OD_600 nm_ was about 0.1) in LB were dispensed into five conical flasks with simultaneous addition of different concentrations of GA. The cultures were continued to incubate at a shaking incubator (180 r/min, 37°C) and the OD_600 nm_ of each conical flask was monitored each hour with a spectrophotometer until reached the plateau phase. The above data were used to plot the growth curve reflecting the interference of GA on the proliferation situation of *K. pneumoniae*.

### 2.5 Time-killing assay

The time-killing assay reflecting the synergistic killing activity of MEM with GA was performed according to the following method (Zhou et al., 2020b). Overnight cultures were diluted and distributed into sterile 96-well microtiter plates at the final concentration of 5 × 10^5^ CFUs/well. Different administrations including GA only, MEM only, combination treatment and blank control were given separately, after which the plate was placed in 37°C incubator for static incubation. At different time points, surviving bacteria were separately coated on LB agar plates for colony counting and spotted on plates for image photography after serial dilutions. In addition, surviving bacteria under different treatments could also be quantified by spectrophotometer.

### 2.6 Checkerboard minimum inhibitory concentration (MIC) determination

The synergistic effect of GA and carbapenems against KPC-2 positive and KPC-2 negative strains was determined using a slightly optimized broth micro-dilution method (Wiegand et al., 2008). Concisely, antibiotics and compounds were serially diluted twofold respectively, and subsequently dispensed into sterile 96-well microtiter plates. Overnight bacteria cultures were diluted and inoculated into plates at the final concentration of 5 × 10^5^ CFUs/well. After 16–18 h of static incubation at 37°C, the turbidity of each well was observed to determine the MICs of antibiotics and compounds for bacteria. The fractional inhibitory concentration (FIC) index values representing the synergistic bactericidal effect of carbapenems and compounds were calculated by the following formula (FIC index ≤ 0.5 implies synergy):


FICindex=MICofcompoundsincombinationMICofcompoundsalone+MICofantibioticsincombinationMICofantibioticsalone

### 2.7 Disk diffusion method

The disk diffusion method was carried out to further determine the synergy of GA and MEM based on previously described (Escamilla-García et al., 2017). Overnight bacteria were diluted and further cultured until reached the exponential growth phase. The tested bacterial suspensions were spread evenly on LB agar plates containing different concentrations of GA. Then the MEM disks (10 μg) were placed in the center of each plate and subsequently incubated at 37°C for 16–18 h. The inhibitory zone diameters were photographed and recorded for data analysis.

### 2.8 *In vitro* live/dead bacteria staining assay

The combined bactericidal effect of GA and MEM was visually evaluated by live/dead bacterial staining assay. Overnight bacterial cultures were diluted in fresh LB broth and then cocultured with GA only [32 μg/mL for *K. pneumoniae* ST-C1 and 8 μg/mL for *E. coli* BL21(DE3) (pET28a-KPC-2)], MEM only [4 μg/mL for *K. pneumoniae* ST-C1 and 1/4 μg/mL for *E. coli* BL21(DE3) (pET28a-KPC-2)] and their combination for 6 h at 37°C. The bacteria were then collected, washed twice and resuspended in sterile PBS, and OD_600 nm_ was adjusted to 0.5. The live or dead status of the tested bacteria of each group was observed with an inverted fluorescence microscope (Nikon Eclipse, Japan) after adding SYTO 9 and propidium iodide (PI) dyes of the LIVE/ DEAD BacLight Bacterial Viability Kit (Invitrogen) under the guidance of the manufacturer’s instructions.

### 2.9 Membrane permeability detection

Alterations in PI and N-Phenyl-1-naphthylamine (NPN) uptake are often used to assess the permeability of inner and outer membranes of bacteria. The bacteria were centrifuged and normalized to the same absorbance value (OD_600 nm_ = 0.5) after treatment with the different drugs (GA only, MEM only, and their combination) for 6 h. PI and NPN were separately added to the suspension at final concentrations of 10 nM and 10 μM and further incubated at 37°C for 90 min. The spectrofluorimeter was used to detect the fluorescence intensity of PI (535 excitation wavelength/615 emission wavelength) and NPN (350 excitation wavelength/420 emission wavelength).

### 2.10 Western blot analysis

Overnight cultures of *K. pneumoniae* were diluted and incubated with GA (0, 8, 32, 128 μg/mL) for 4 h or 8 h at 37°C. Then the cultures were collected, standardized and prepared as samples for western blot assay after boiled at 100°C for 10 min. After separated by SDS-PAGE (12% gels), electrophoresed proteins were transferred onto a polyvinylidene difluoride (PVDF) membrane. The membranes were then blocked with 5% skim milk for 2 h at room temperature, followed by successive incubation with primary antibodies against KPC-2 (prepared from mouse) and goat anti-mouse IgG secondary antibody (HRP). ICDH was used as an internal reference. Finally, the targeted protein was visualized with an enhanced chemiluminescence substrate.

### 2.11 Circular dichroism spectra detection

Circular dichroism (CD) spectra was measured using a CD spectrophotometer (MOS-500; Bio-Logic) with the wavelength ranging from 190 to 250 nm at ambient temperature. The recorded spectra were subsequently analyzed with *BeStSel web server* to compare the alterations in the secondary structure of KPC-2 proteins with or without GA treatment.

### 2.12 Fourier transform infrared spectroscopy analysis

Fourier transform infrared spectroscopy (FTIR) spectrometer was used to record the FTIR spectra of KPC-2 protein treated with or without 32 μg/mL of GA over a range of 4,000–500 cm^−1^ with a resolution of 2 cm^−1^. The obtained data was analyzed and graphed with Origin 2023, revealing the effect of GA treatment on the functional groups of the proteins.

### 2.13 Bacterial nucleic acid and protein leakage

The nucleic acid and protein leakage detection was measured following a previously described method with modifications (Yao et al., 2014). Bacteria in logarithmic phase were centrifuged at low speed (≤5,000 r/min) and then resuspended in PBS (pH 7.2). After adjusting the OD_600 nm_ to 0.5, bacterial suspensions were partitioned and different concentrations of GA were added. Subsequently, a 4-h culture was conducted at a shaking incubator (180 r/min, 37°C). At different time points, 1 mL of bacterial culture from each group was collected and the protein and nucleic acid concentrations in the supernatant were detected using a DNA/protein analyzer.

### 2.14 Molecular docking and molecular dynamics simulation

Molecular docking was performed with autodock vina1.1.2 software to simulate the binding modes of the KPC-2-GA complex and KPC-2-MEM complex (Tsang et al., 2022), before which the three-dimensional (3-D) structures of GA, MEM, and KPC-2 were downloaded from RCSB and pubchem, respectively. The amber18 software was then used to conduct MD simulations of the KPC-2-GA complex and KPC-2-MEM complex (Arodola et al., 2020), during which ff14SB forcefield parameter was used for proteins and gaff generic forcefield parameter was used for compound and antibiotics. The essential protein residues for ligand-protein binding were specified by decomposing the binding free energy of the complexes with the molecular mechanics Poisson-Boltzmann surface area (MM-PBSA) approach (Rajeshkumar et al., 2022).

### 2.15 Statistical analysis

All the assays were conducted at least three biological replicates and the data were expressed as the mean ± standard deviation. Statistical analysis was calculated by Student’s *t* test for two groups and one-way analysis of variance (ANOVA) for multiple groups in GraphPad Prism 9.5.1. A value of *p* < 0.05 was regarded as statistically significant.

## 3 Results

### 3.1 GA attenuated the enzymatic activity of KPC-2 without affecting bacterial viability and KPC-2 expression

**Figure 1:**
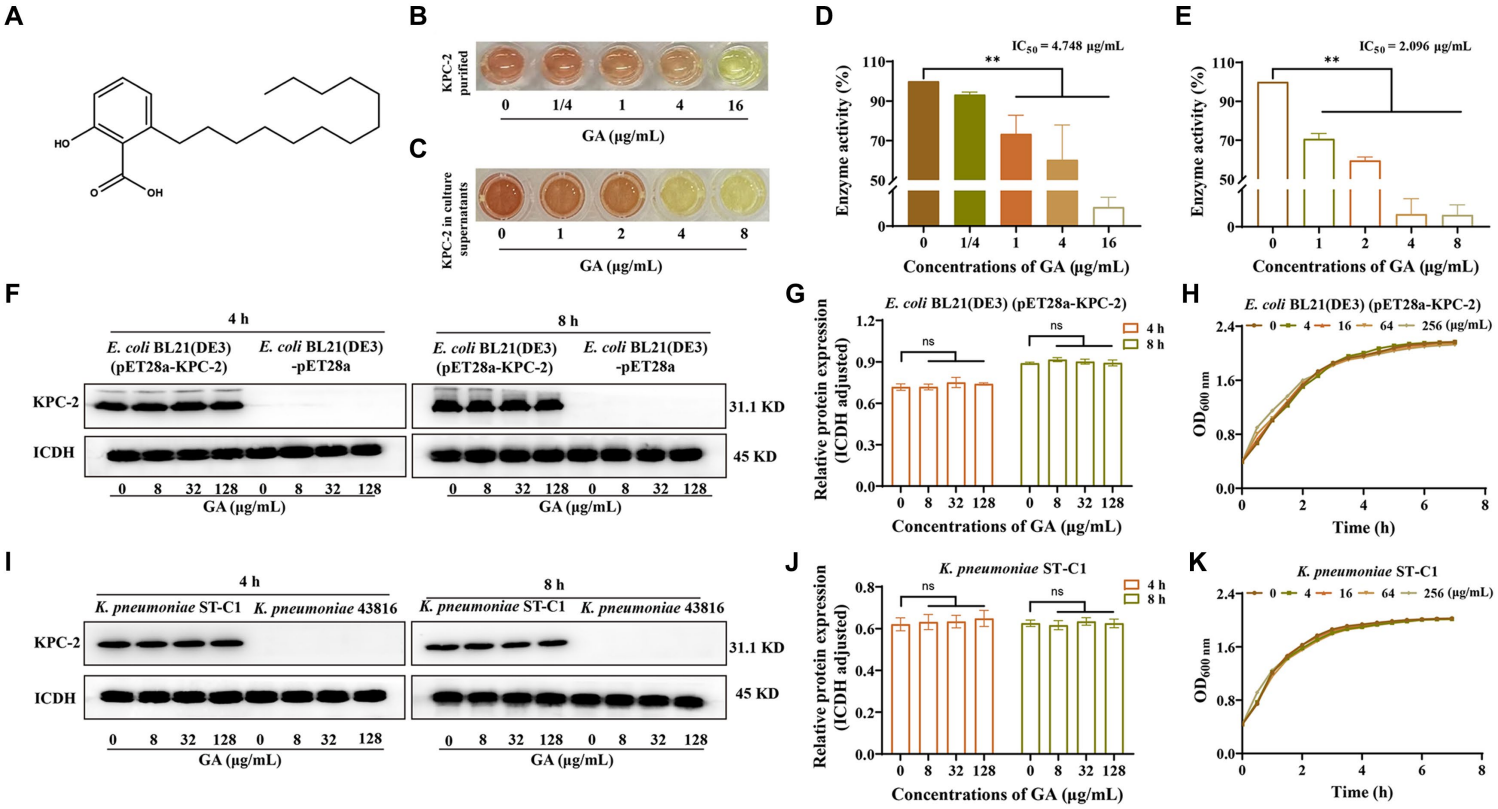
GA inhibited the activity but not the expression of KPC-2. **(A)** Chemical structure of GA. The inhibitory effect of GA on purified KPC-2 protein **(B,D)** and KPC-2 in culture supernatants **(C,E)** was manifested by a color change and enzyme activity quantification. Effects of multiple concentrations of GA on KPC-2 expression in *E. coli* BL21(DE3) (pET28a-KPC-2) **(F,G)** and *Klebsiella pneumoniae* ST*-*C1 **(I,J)**. The growth curves of *E. coli* BL21(DE3) (pET28a-KPC-2) **(H)** and *K. pneumoniae* ST*-*C1 **(K)** co-cultured with several concentrations of GA (0/4/16/64/256 μg/mL).

Enzyme inhibition assays were used to assess the alterations in KPC-2 activity induced by different compounds. GA (Figure 1A) was ultimately identified as a potent KPC-2 inhibitor, manifested by a dose-dependent reduction in the activity of the purified protein (Figures 1B,D) and protein secreted into culture medium (Figures 1C,E). The IC_50_ of GA for KPC-2 inhibition were 4.748 μg/mL and 2.096 μg/mL, respectively.

We next explored the effect of GA on the expression of KPC-2 protein. As shown in Figures 1F,G,I,J, GA had no disturbing effect on KPC-2 production in *E. coli* BL21(DE3) (pET28a-KPC-2) and *K. pneumoniae* ST*-*C1. Moreover, we demonstrated that different concentrations of GA (0–128 μg/mL) had no visible inhibitory efficacy on the growth of KPC-2-positive bacteria (Figures 1H,K). Taken together, the above results suggested that GA at non-bactericidal concentrations could significantly inhibit the activity of KPC-2 without affecting KPC-2 expression.

### 3.2 GA re-sensitized KPC-2 positive *Klebsiella pneumoniae* to carbapenems

**Figure 2:**
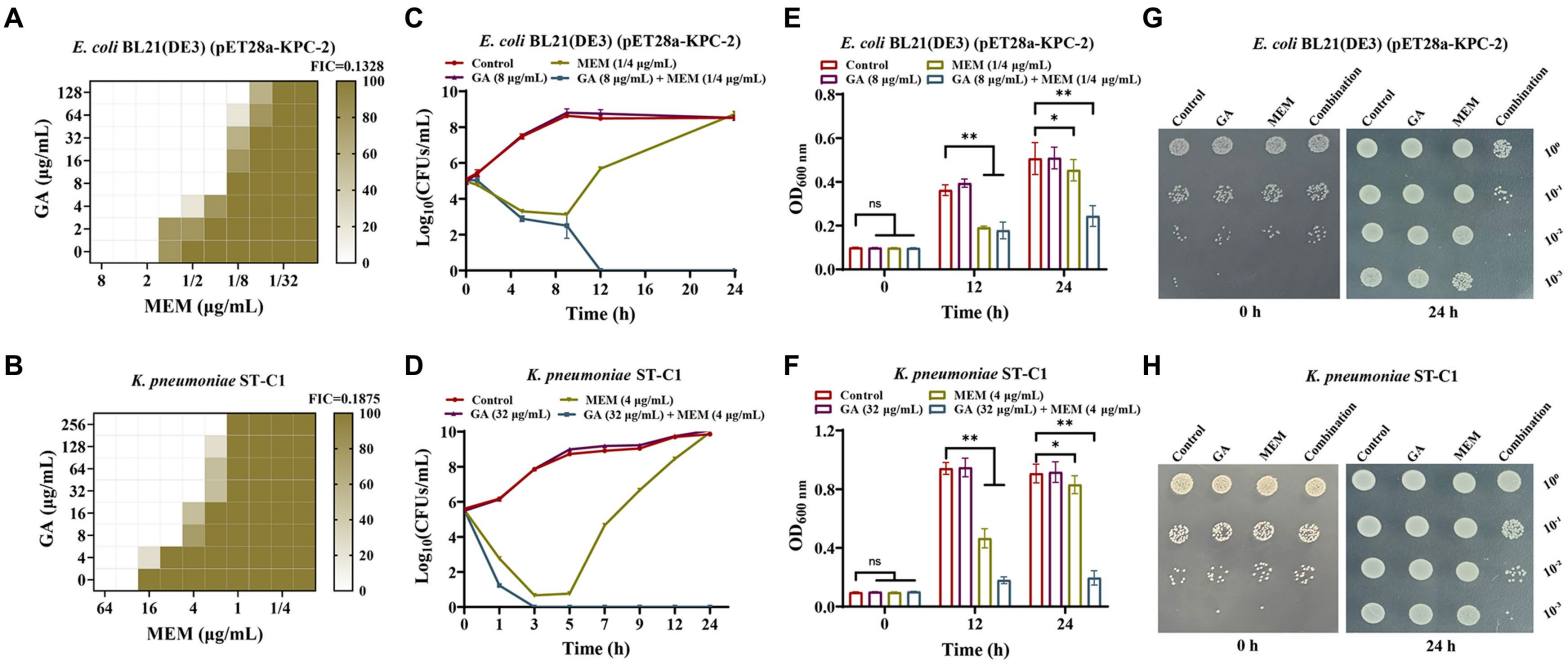
GA synergised with MEM to kill KPC-2 positive bacteria. **(A,B)** Microdilution checkerboard analysis was implemented to assess the synergistic effect of MEM with GA against *E. coli* BL21(DE3) (pET28a-KPC-2) and *Klebsiella pneumoniae* ST*-*C1. Time-killing curves for *E. coli* BL21(DE3) (pET28a-KPC-2) **(C)** and *K. pneumoniae* ST-C1 **(D)** when treated with GA, MEM, combination and medium only. OD_600 nm_ values reflecting the survival *E. coli* BL21(DE3) (pET28a-KPC-2) **(E)** and *K. pneumoniae* ST-C1 **(F)** in the combined treatment of GA and MEM. The spot assays of *E. coli* BL21(DE3) (pET28a-KPC-2) **(G)** and *K. pneumoniae* ST-C1 **(H)** on LB agar plates were performed after treated with different drugs (GA, MEM, combination and medium only) for 0 h and 24 h. After serial dilutions, the cultures were dropped onto plates and incubated overnight at 37°C.

The activity inhibition of GA on KPC-2 prompted us to verify the synergistic effect between GA and carbapenems. The checkboard MIC assays showed that the MIC of MEM against *E. coli* BL21(DE3) (pET28a-KPC-2) and *K. pneumoniae* ST-C1 could be down-regulated ≥8-fold by the combination of GA (FIC index < 0.2; Figures 2A,B). GA also exhibited significant synergistic effects with imipenem (Supplementary Figures S1A,B). However, in carbapenem-sensitive strains without KPC-2, this synergistic effect could not be observed (Supplementary Figures S1C–F). The synergistic bactericidal activity of GA with MEM was further demonstrated using the time-killing test. Remarkably, combination therapy killed more of the tested bacteria within 24 h in both laboratory strain *E. coli* BL21(DE3) (pET28a-KPC-2; Figures 2C,E,G) and clinical strain *K. pneumoniae* ST*-*C1 (Figures 2D,F,H).

**Figure 3:**
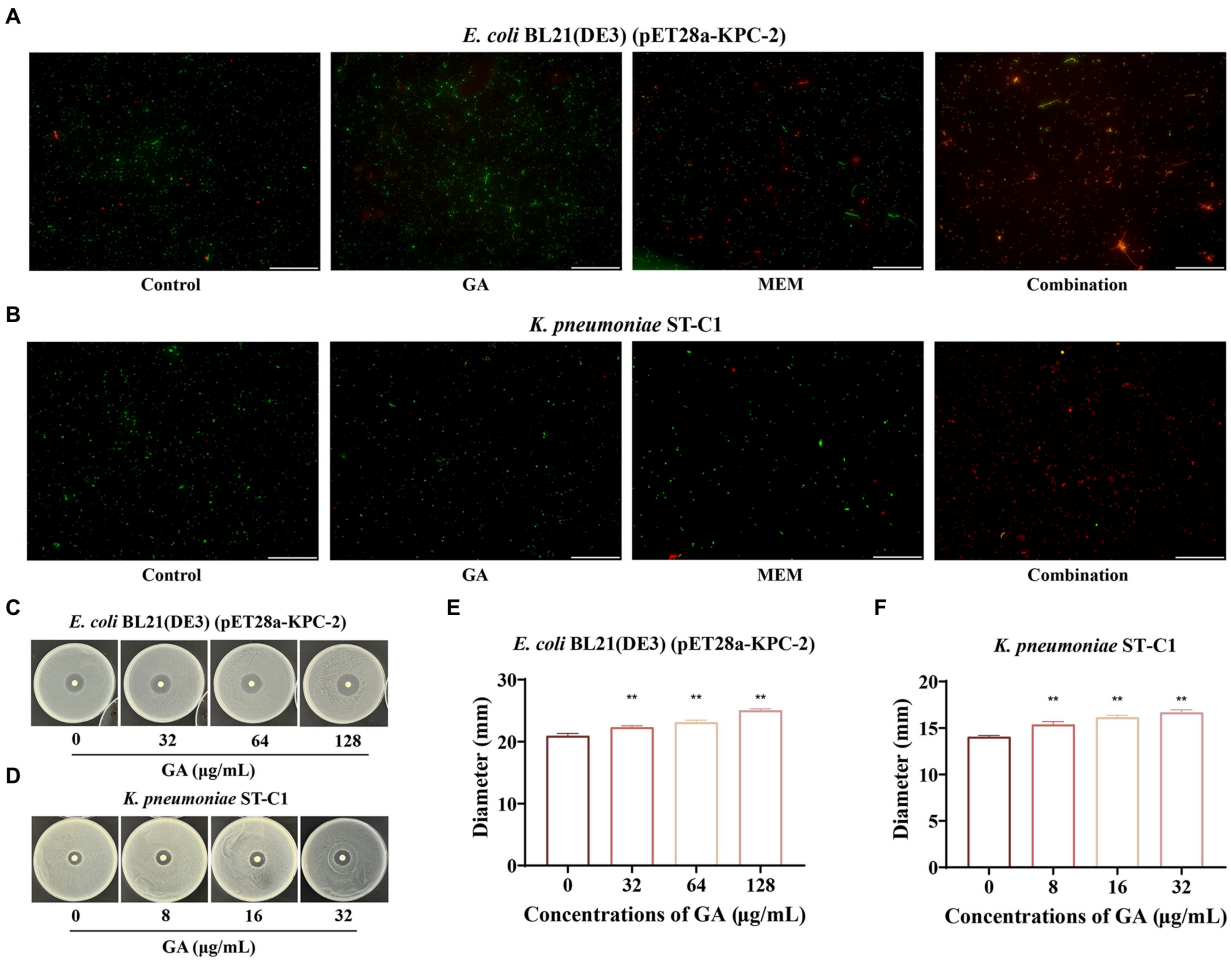
GA restored the bactericidal activity of MEM against KPC-2 positive bacteria. The live/dead bacteria staining for *E. coli* BL21(DE3) (pET28a-KPC-2) **(A)** and *Klebsiella pneumoniae* ST-C1 **(B)** after treatment with GA, MEM or GA plus MEM (scale bar = 200 μm). The zone diameters surrounding MEM disks were expanded by GA in a dose-dependent manner on LB agar plates coated with *E. coli* BL21(DE3) (pET28a-KPC-2) **(C,E)** and *K. pneumoniae* ST-C1 **(D,F)**.

The live/dead bacteria staining assay and combined disk test were then successively used to visually compare the bactericidal ability of monotherapy with that of combination therapy. Figures 3A,B showed that GA combined with MEM extremely exacerbated bacterial death, as evidenced by a higher ratio of dead (red) to alive bacteria (green). Consistent with these results, the diameters of the inhibitory zones of the tested bacteria surrounding the MEM disks increased in a dose-dependent manner with GA concentration (Figures 3C–F). Overall, these findings illustrated that GA and MEM had a favorable synergistic effect against KPC-2-positive bacteria.

### 3.3 GA altered the secondary structure and functional groups of KPC-2 through direct engagement

**Figure 4:**
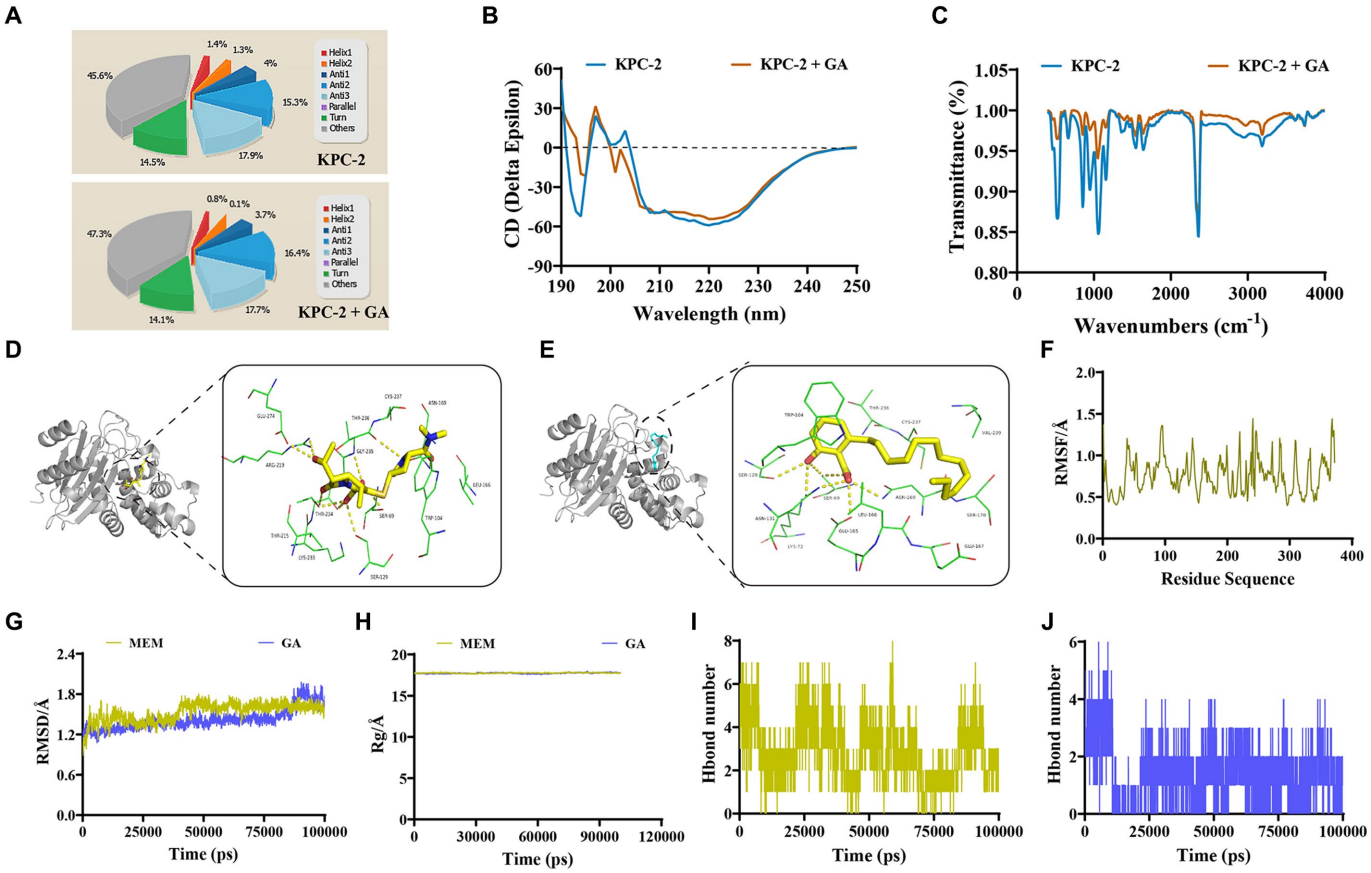
Identification of the mechanism by which GA inhibited KPC-2 activity. **(A,B)** Secondary structure changes of KPC-2 in the presence or absence of GA (32 μg/mL) were detected by CD spectroscopy. **(C)** GA (32 μg/mL)-induced alteration of the KPC-2 functional group was confirmed by FTIR assay. The 3-D binding modes of MEM **(D)** and GA **(E)** to KPC-2 were predicted by MD simulations. **(F)** RMSF image indicating the fluctuation of amino acid residues of KPC-2 throughout the entire kinetic process. RMSD **(G)** and Rg **(H)** detection of the MEM-KPC-2 complex and GA-KPC-2 complex throughout the simulation. Hydrogen bond numbers of MEM **(I)**, GA **(J)** conjugated to KPC-2 during the MD simulation.

To initially characterize the molecular basis for the inhibition of KPC-2 activity by GA, CD spectra was first performed to compare the differences in the secondary structure of KPC-2 with or without GA treatment. A visible conformational change was observed in GA-induced KPC-2, characterized by decreased a-helix1, a-helix2, and elevated anti-2 conformation (Figures 4A,B). FTIR also revealed that GA could alter the composition and ratio of the functional groups of KPC-2, further supporting the above results (Figure 4C).

The specific binding modes between KPC-2 with GA or MEM were subsequently explored by a computational biological method called molecular docking and MD simulation. During the entire kinetic process, the amino acid residues of the KPC-2 protein do not fluctuate dramatically (Figure 4F), and both GA and MEM bound stably to the KPC-2 protein without causing sustained changes in protein conformation and compactness (Figures 4G,H). The 3-D binding model showed that both GA and MEM bound within the catalytic pocket of KPC-2 and the conformational superimpositions of the two small molecules were highly overlapping (Figures 4D,E). Although the binding energy of MEM to KPC-2 (−8 kcal/moL) was slightly higher than that of GA (−6.5 kcal/moL), the numerous common binding sites and the similar number of hydrogen bonds (Figures 4I,J) still indicated that GA was strongly competitive for MEM binding to KPC-2.

**Figure 5:**
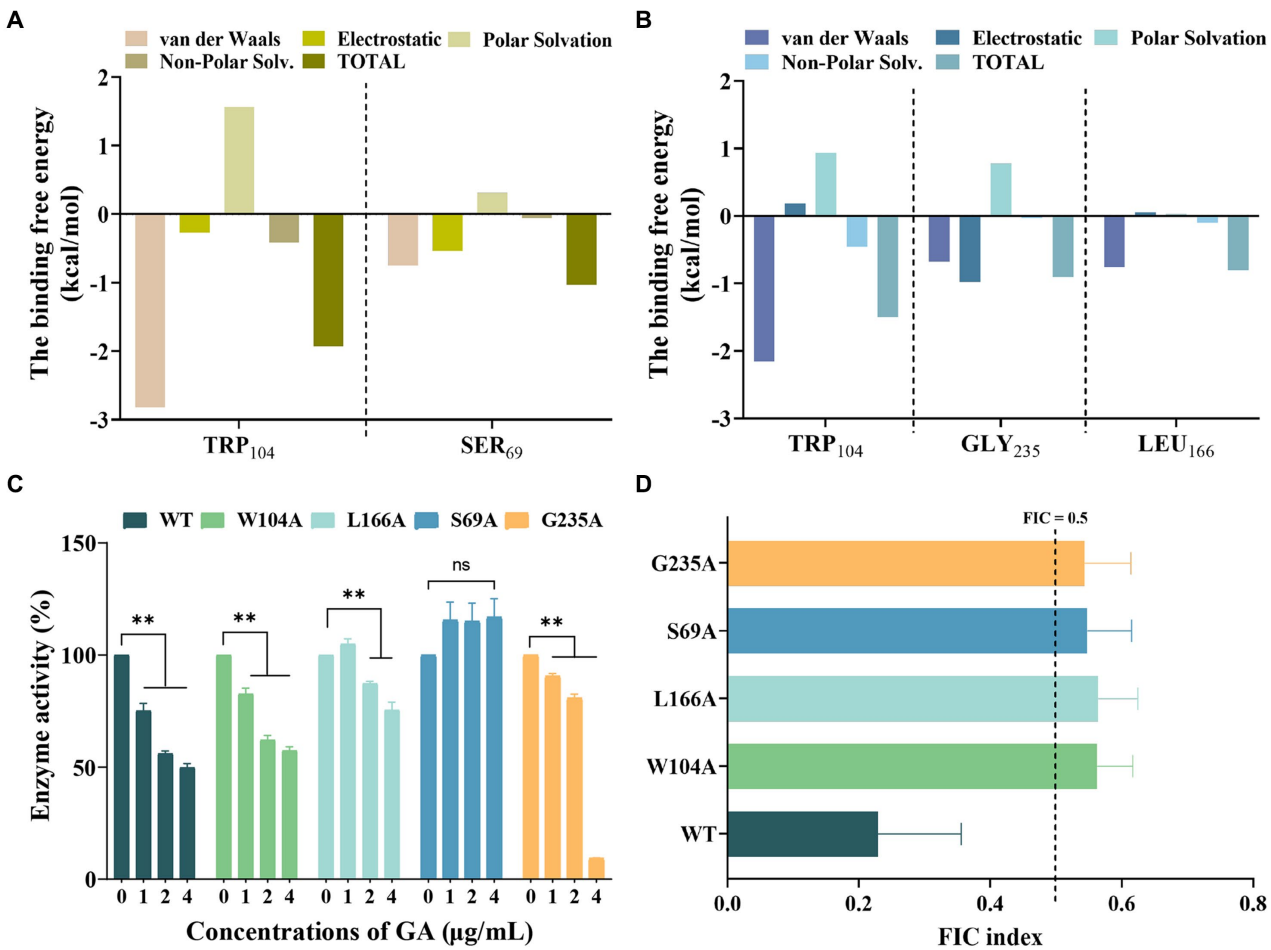
Confirmation of the binding sites of KPC-2 with MEM and GA. Decomposition of the binding energy on a per-residue basis in the KPC-2-MEM **(A)** and KPC-2-GA complex **(B)**. **(C)** Influence of GA on the enzymatic activity of KPC-2 and its mutants. **(D)** FIC index values reflecting the synergistic effect of GA and MEM against each of the variants.

The total binding free energies and the detailed energy contributions for the KPC-2-GA and KPC-2-MEM complexes were calculated using the MM-PBSA approach. The results of energy decomposition revealed that residues Trp_104_, and Ser_69_ had powerful contributions to the KPC-2-MEM complex (ΔE_total_ of ≤ −1 kcal/mol; Figure 5A), while residues Trp_104,_ Gly_235,_ and Leu_166_ formed strong interactions with GA (ΔE_total_ of ≤ −0.8 kcal/mol; Figure 5B). To verify the reliability of the simulated results, we performed targeted mutagenesis of the above amino acid sites contributed crucially, after which we repeated the enzyme inhibition assay and the MIC test in samples containing mutated KPC-2. Obviously, GA lost the significant enzyme activity inhibitory effect against mutant KPC-2 (Figure 5C). A similar reduction in the synergistic ability of GA with MEM against strains containing mutated KPC-2 was also observed (Figure 5D). In brief, our results illustrated that GA competitively bound to KPC-2, altered its secondary structure and functional groups, ultimately realizing its enzyme activity inhibitory efficacy.

### 3.4 GA potentiated the disruptive effect of carbapenems on bacterial membranes

**Figure 6:**
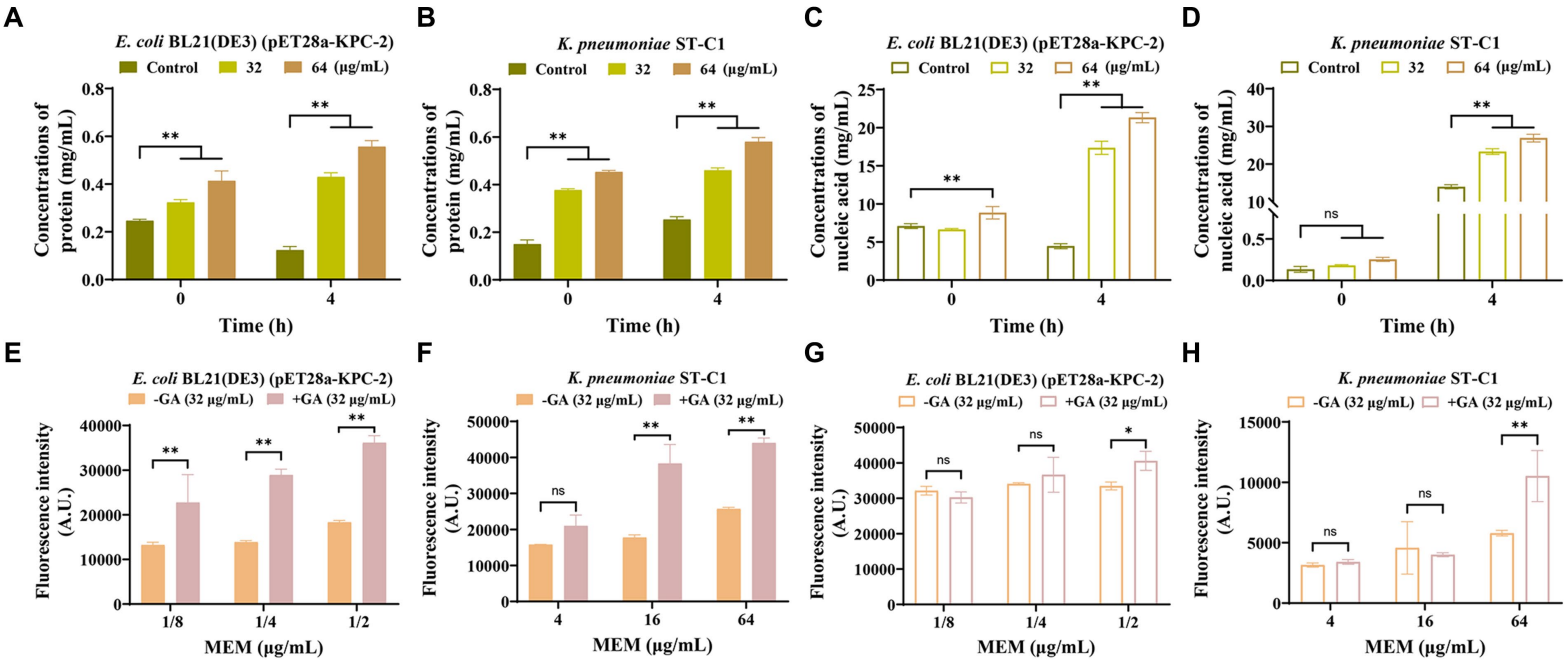
GA strengthened the disrupting effect of MEM on bacterial membranes. GA induced the protein and nucleic acid leakage of *E. coli* BL21(DE3) (pET28a-KPC-2) **(A,C)** and *Klebsiella pneumoniae* ST-C1 **(B,D)** in a dose-dependent and time-dependent manner. Increased permeability of the inner membrane of *E. coli* BL21(DE3) (pET28a-KPC-2) **(E)** and *K. pneumoniae* ST-C1 **(F)** by a combination treatment with GA. PI was used as the probe at a final concentration of 10 nM. The outer membrane permeability of *E. coli* BL21(DE3) (pET28a-KPC-2) **(G)** and *K. pneumoniae* ST-C1 **(H)** could be enhanced when synergized with GA compared to MEM alone.

Considering that targeting membranes is the main mechanism by which carbapenems function, we speculated whether GA alone or in concert with MEM could aggravate membrane damage. We first explored the capacity of GA on membrane damage by measuring GA-induced nucleic acid and protein leakage. The results showed a dose- and time-dependent increase in the levels of nucleic acids and proteins released into the supernatant by the strains co-cultured with GA (Figures 6A–D).

The fluorescent probes PI and NPN were subsequently used to assess the synergistic effect of GA with MEM in disrupting bacterial membranes. As shown in Figures 6E–H, co-incubation with GA significantly increased the ability of PI and NPN to enter the bacteria compared to the control group, indicating the potent effect of GA in enhancing the permeability of both the inner and outer membranes, especially the inner membrane. Hence, we could infer that disturbing membrane integrity may be another pathway for GA to synergize with carbapenems.

## 4 Discussion

The emergence and prevalence of CRKP coupled with the lag in antibiotic development have driven the ongoing search for strategies to enhance the efficacy of existing carbapenems. Among these strategies, the identification of potent inhibitors targeting KPC-2 thereby restoring the bactericidal capacity of carbapenems has become a prominent area of research (Zhou et al., 2020b). We were pleased to uncover that GA could inhibit the enzymatic activity of KPC-2 by directly binding and then altering its secondary structure and functional groups. Concurrently, GA could also synergize with carbapenems to disrupt bacterial membranes, as evidenced by elevated nucleic acid and protein leakage and increased membrane permeability. Of interest, the synergistic effect of GA with carbapenems is indeed present in both laboratory strains and clinical strains, suggesting the potential of GA for clinical therapeutic.

*Ginkgo biloba* (Ginkgo), which is known as a “living fossil,” has been documented as a medicinal plant 2,800 years ago (Jacobs and Browner, 2000). Its extracts have been reported to possess multiple pharmacological and clinical efficacy (Maitra et al., 1995; Zhang et al., 2008; Mashayekh et al., 2011; Hamdoun and Efferth, 2017; Yang et al., 2017; Zheng et al., 2021; Kim et al., 2022) and are the most widely used herbal medicines and dietary supplements worldwide (Ngan et al., 2012). Currently, research on GA, an extract of *Ginkgo biloba*, is focused mainly on cancer treatment as well as diabetes control. In our study, we proposed that GA possessed a significant effect against KPC-2-positive *K. pneumoniae*. Concerning *in vivo* applications, we already predicted the ADMET profile of GA using SwissADEM and ADMETlab servers. The results indicated that GA is easily absorbed through the gastrointestinal tract and has a well bioavailability efficiency (Table 1). It is noteworthy that GA was not predicted to have significant cellular and *in vivo* toxicity. In the near future, the dosing regimen and exact *in vivo* efficacy of GA need to be further explored to optimize its effects.

**Table 1:** ADMET profile of ginkgolic acid predicted by SwissADEM and AD METlab servers.

Absorption		Distribution		Metabolism		Excretion		Toxicity	
GI absorption	High	BBB permeant	No	CYP1A2 inhibitor	Yes	CLplasma	5.045	AMES Toxicity	0.163
Caco-2 Permeability	−4.71	VSss	1.641	CYP2C19 inhibitor	Yes	T1/2	0.446	Rat Oral Acute Toxicity	0.275
Pgp substrate	No	Fu	6.30%	CYP2C9 inhibitor	Yes			Carcinogenicity	0.195
Bioavailability Score	0.85	BCRP inhibitor	No	CYP2D6 inhibitor	No			A549 Cytotoxicity	0.016

In summary, our results demonstrated that GA could strengthen the bactericidal effect of carbapenems against CRKP by simultaneously inhibiting KPC-2 activity and damaging bacterial membranes. The combination of carbapenems and GA may be a promising alternative strategy against KPC-2-containing *K. pneumoniae.* The next step in our study will be to examine the efficacy of GA in combination with carbapenems in the animal model of KPC-2-positive *K. pneumoniae* infection.

## Data Availability

The original contributions presented in the study are included in the article/Supplementary material, further inquiries can be directed to the corresponding authors.
