# Supplementary material for: Ginkgolic Acid as a carbapenem synergist against KPC-2 positive Klebsiella pneumoniae
Source: Front Microbiol. 2024 Aug 21;15:1426603. doi: 10.3389/fmicb.2024.1426603 (PMC11371739; doi:10.3389/fmicb.2024.1426603)
Supplement: Supplementary file 1 [file Data_Sheet_1.docx]

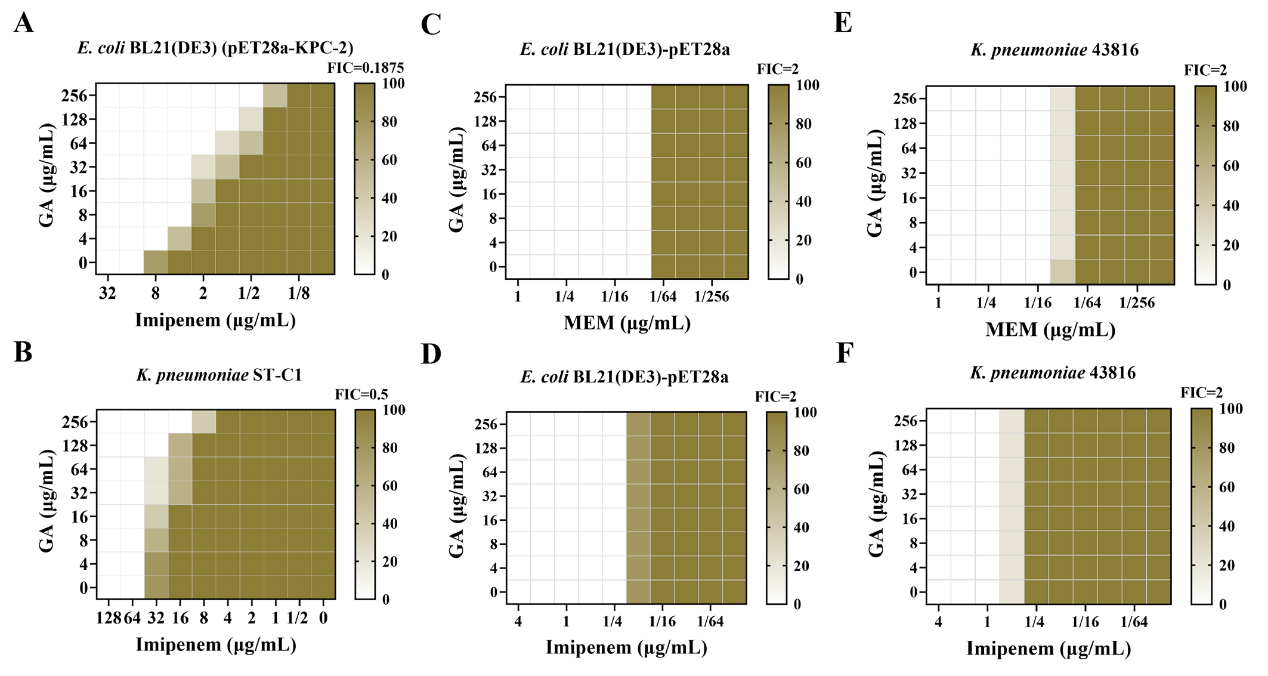


**Supplementary Figure 1**

Synergistic effect of GA with carbapenems against bacteria with or without KPC-2. GA exhibited a synergistic effect with imipenem against *E. coli* BL21(DE3) (pET28a-KPC-2) **(A)** and *Klebsiella pneumoniae* ST-C1 **(B)**. GA showed no synergistic effect with carbapenems in *E. coli* BL21(DE3)-pET28a and *Klebsiella pneumoniae* 43816 strains without KPC-2 **(C-F)**.
