# Supplementary material for: Ginkgolic Acid as a carbapenem synergist against KPC-2 positive Klebsiella pneumoniae
Source: Front Microbiol. 2024 Aug 21;15:1426603. doi: 10.3389/fmicb.2024.1426603 (PMC11371739; doi:10.3389/fmicb.2024.1426603)
Supplement: Supplementary file 2 [file Table_1.docx]

**Supplementary Table 1** Sequence of primers used for site-directed mutagenesis

| **Gene** | **Primer** | **Sequence (5´-3´)** |
| --- | --- | --- |
| KPC-2_W104A_ | Forward | CGCTGGTTCCGGCGTCACCCATCTC |
|  | Reverse | GAGATGGGTGACGCCGGAACCAGCG |
| KPC-2_L166A_ | Forward | GACCGCTGGGAGGCGGAGCTGAACTC |
|  | Reverse | GAGTTCAGCTCCGCCTCCCAGCGGTC |
| KPC-2_S69A_ | Forward | CGCTTCCCACTGTGCGCGTCATTCAAGGGCTTTC |
|  | Reverse | GAAAGCCCTTGAATGACGCGCACAGTGGGAAGCG |
| KPC-2_G235A_ | Forward | GAGACAAAACCGCGACCTGCGGAGTG |
|  | Reverse | CACTCCGCAGGTCGCGGTTTTGTCTC |
